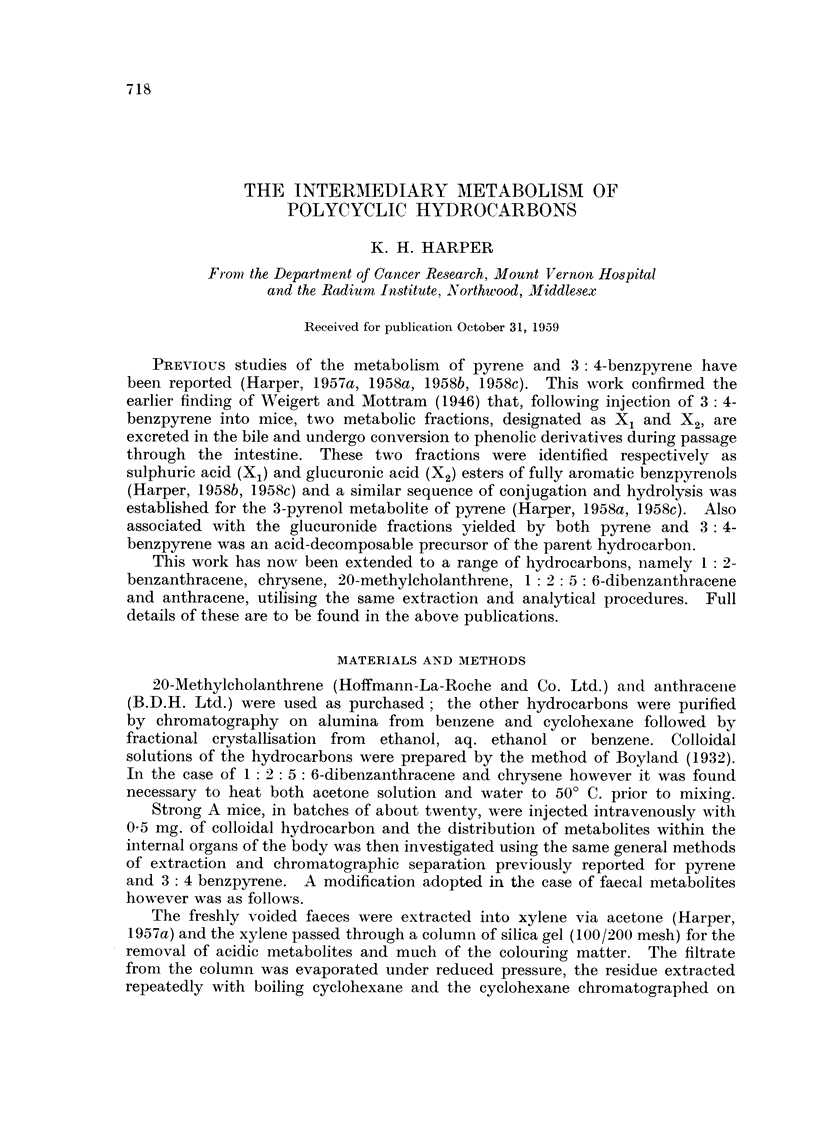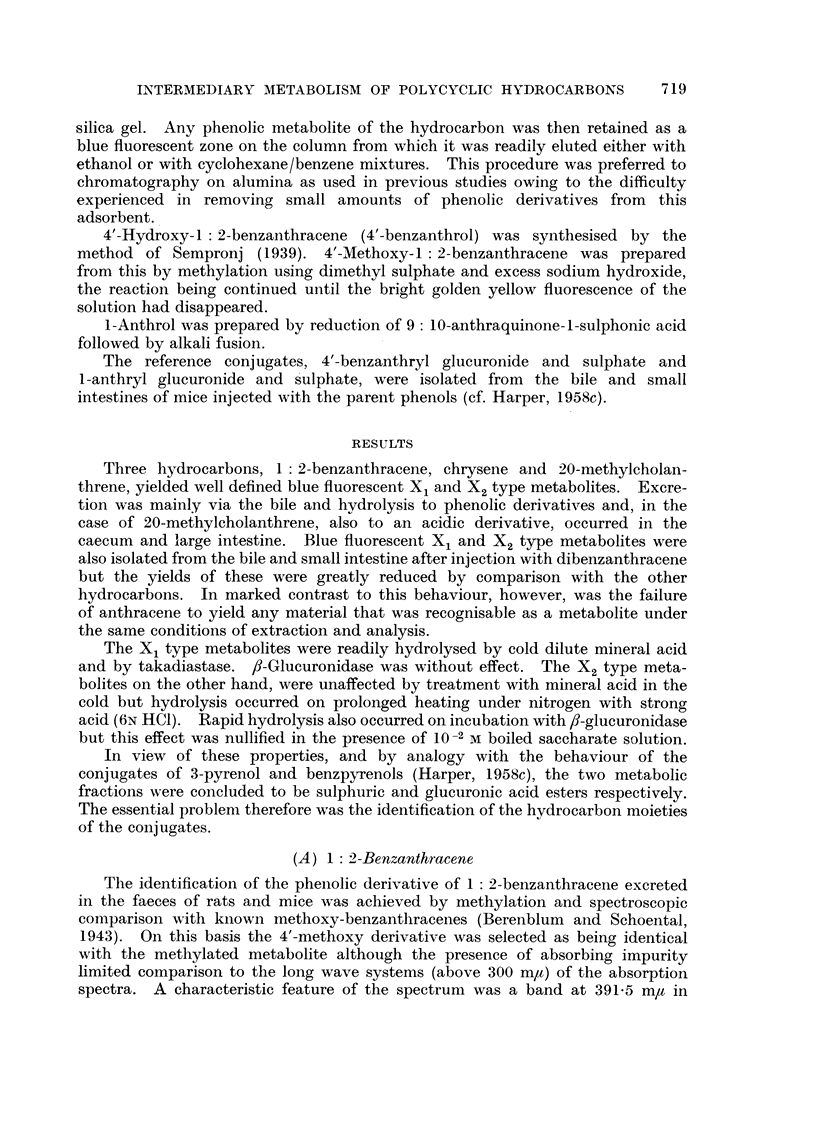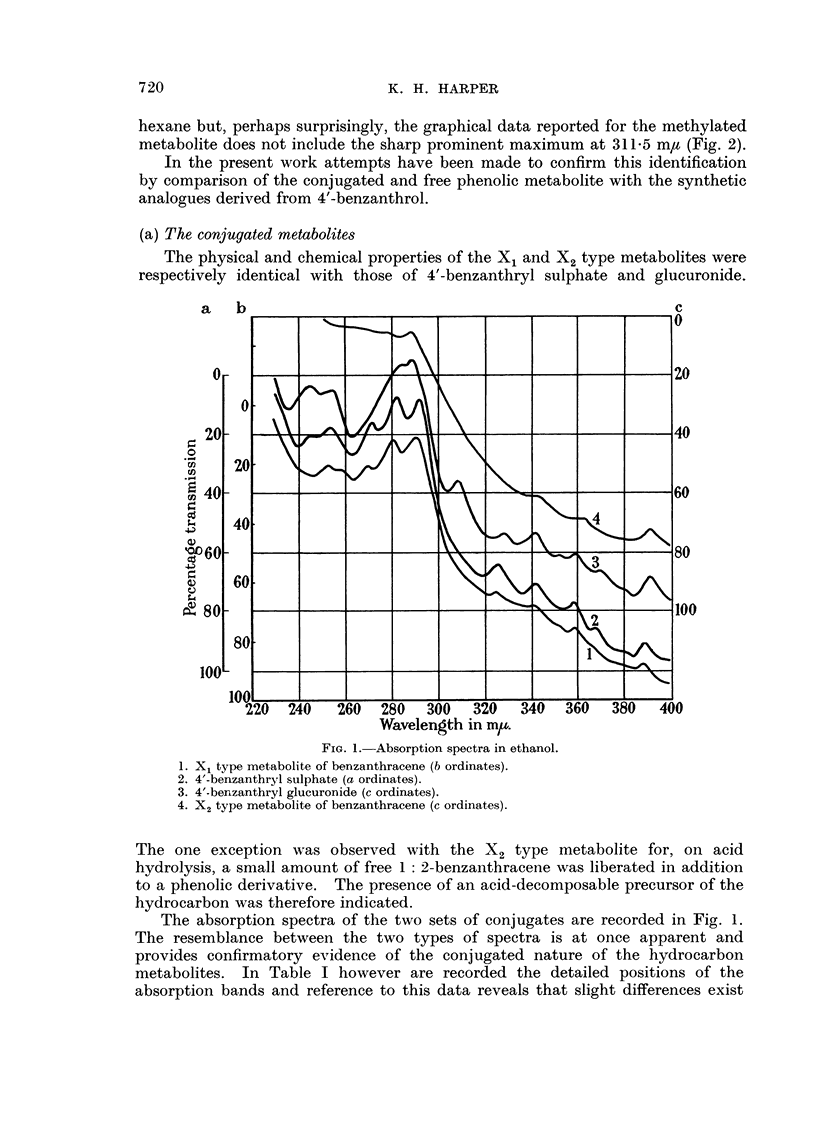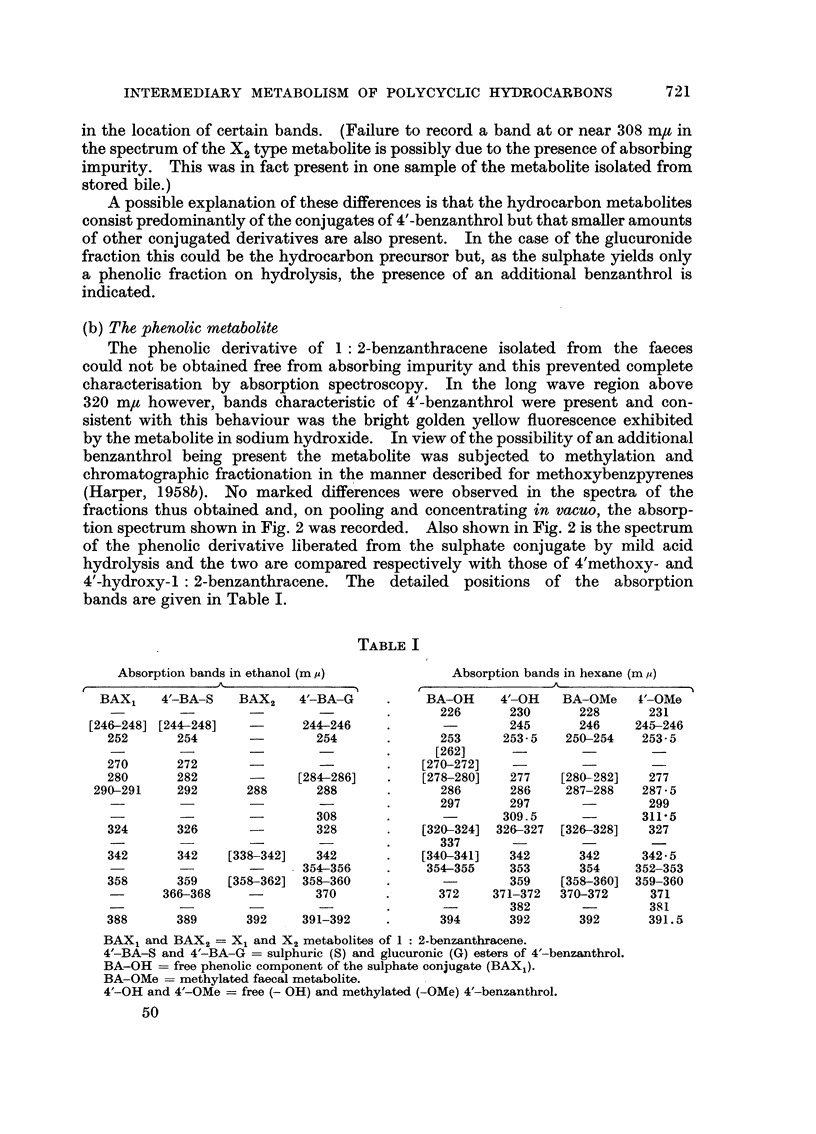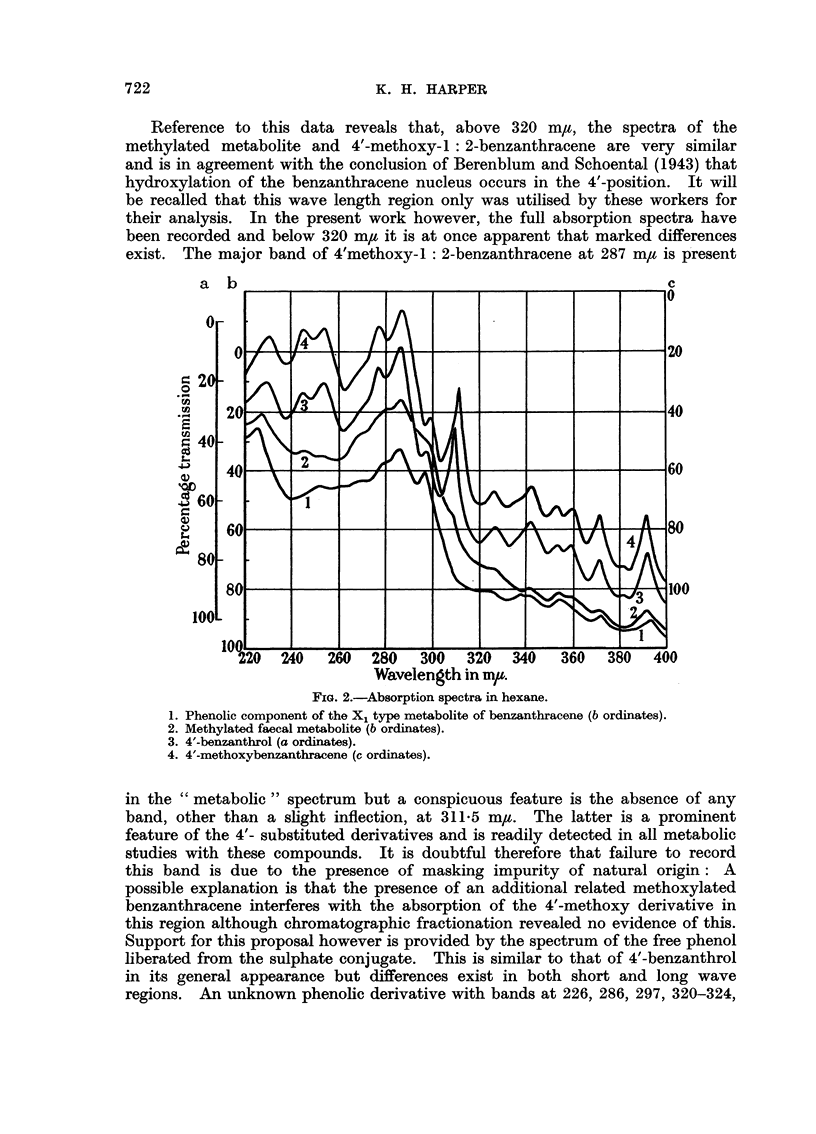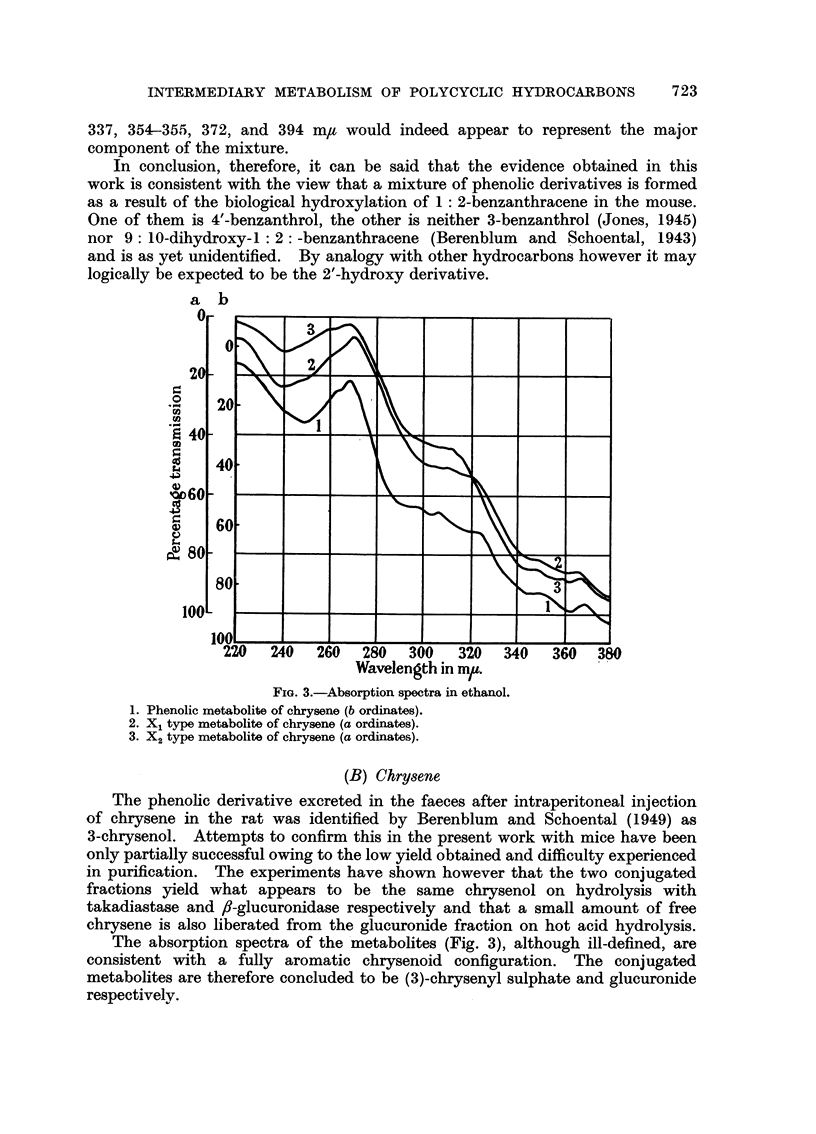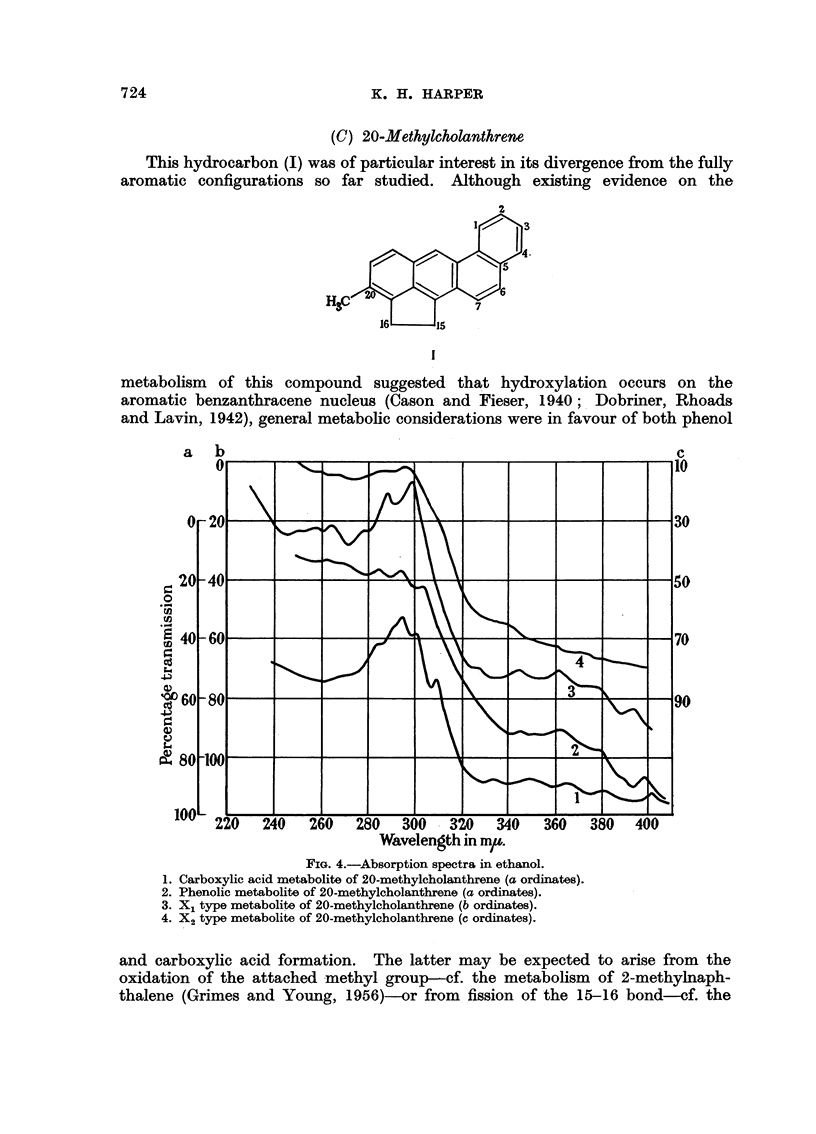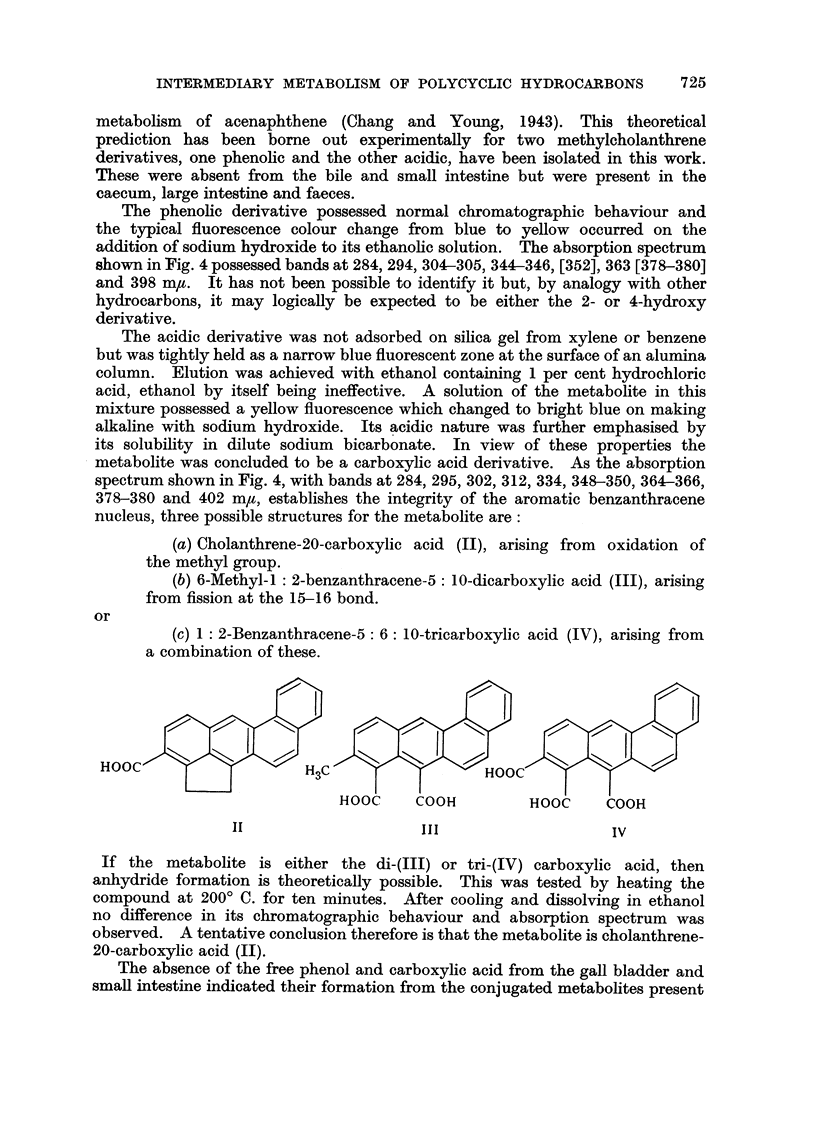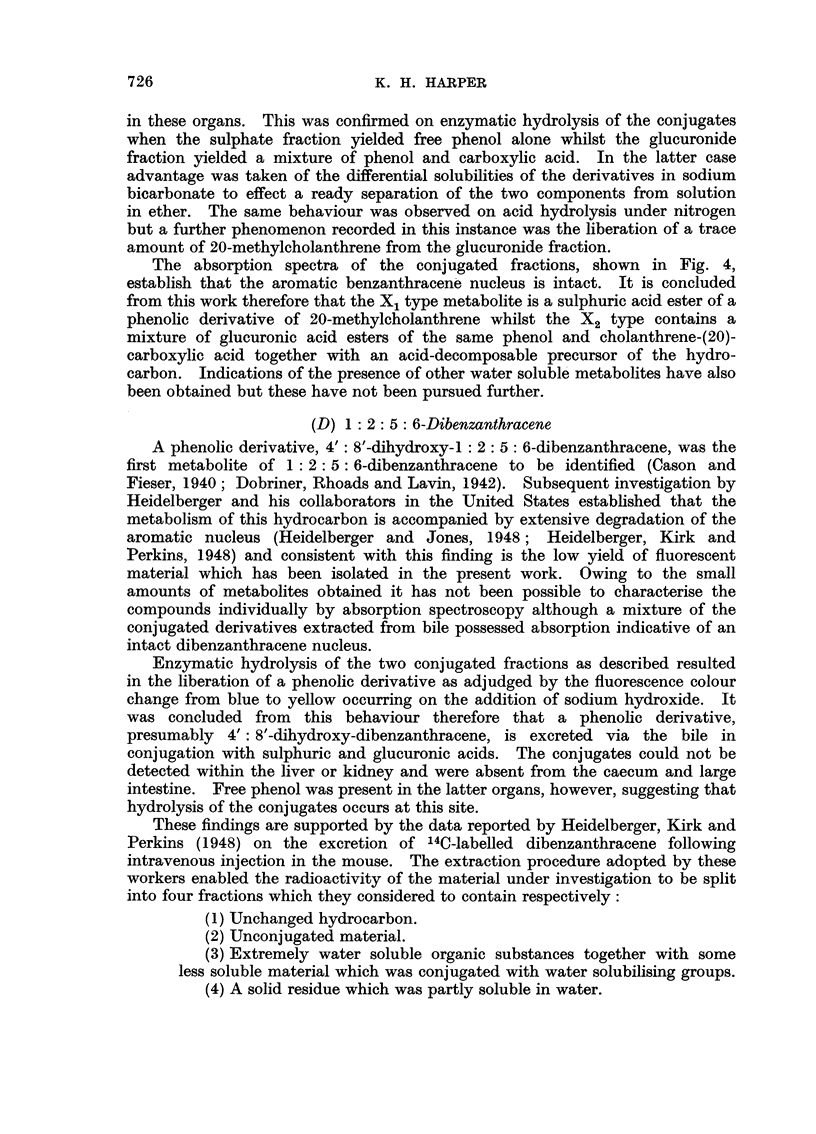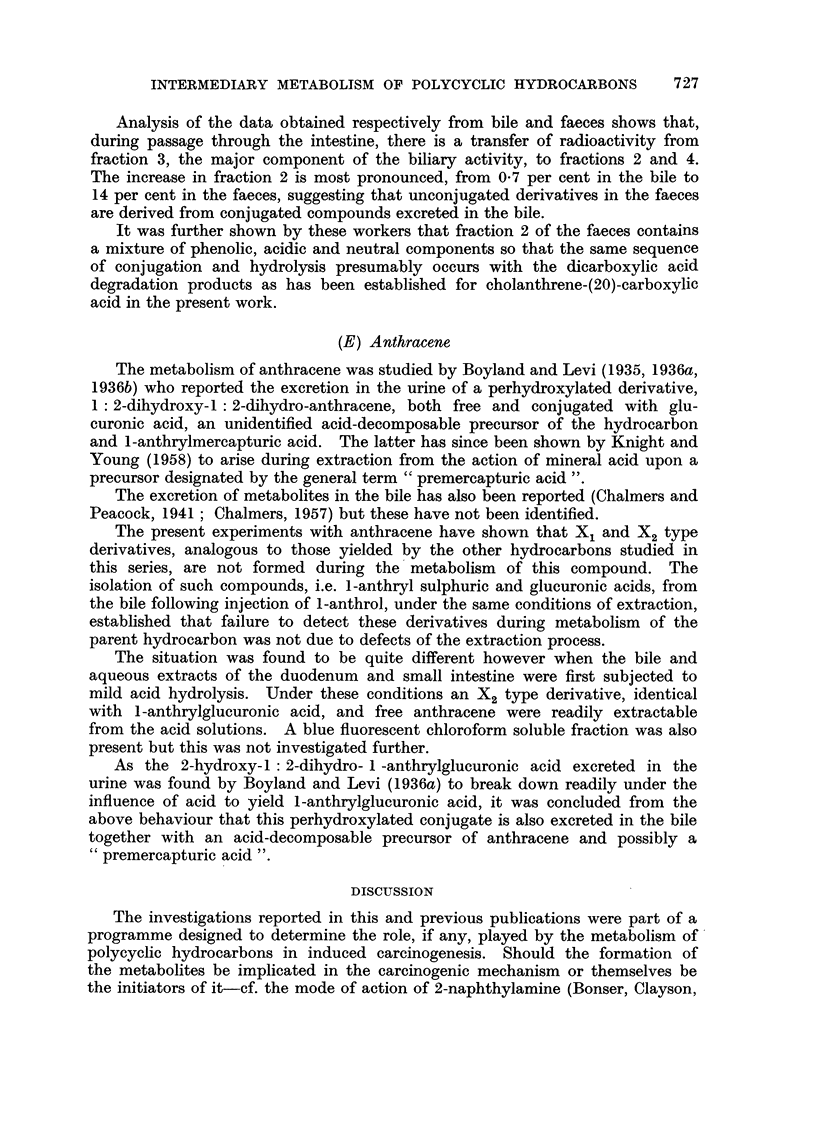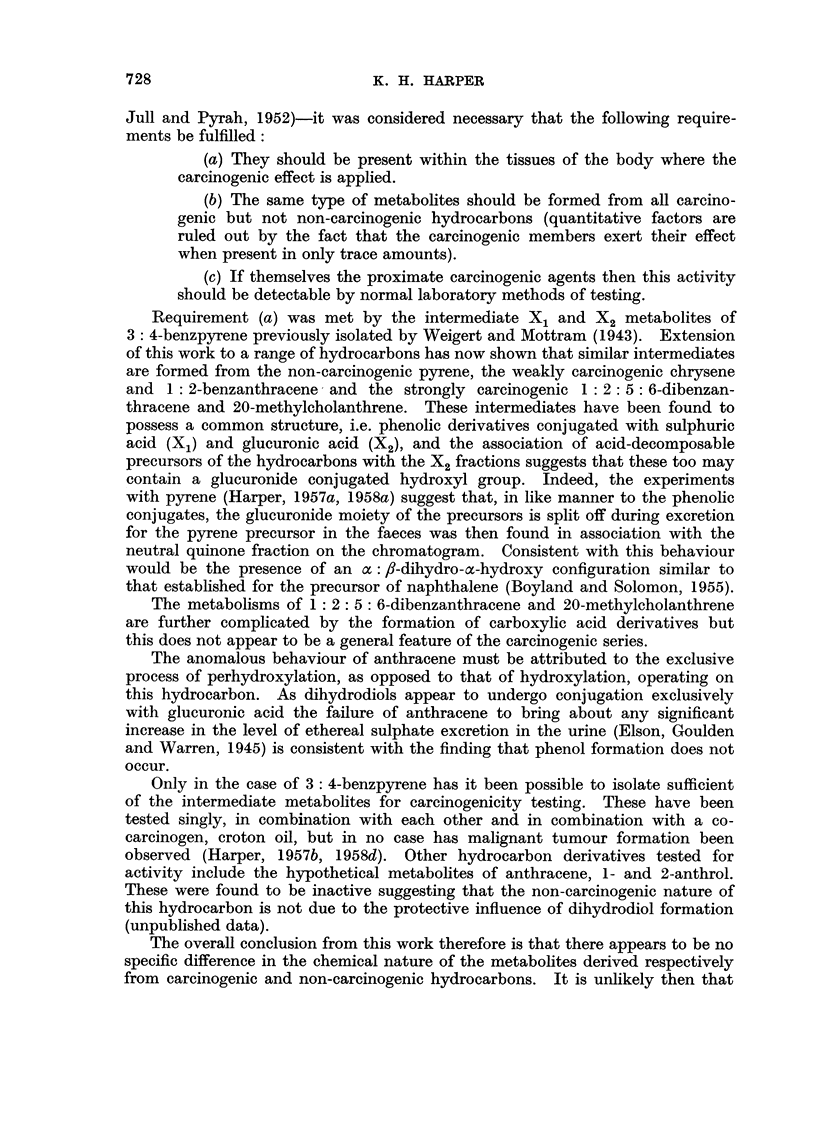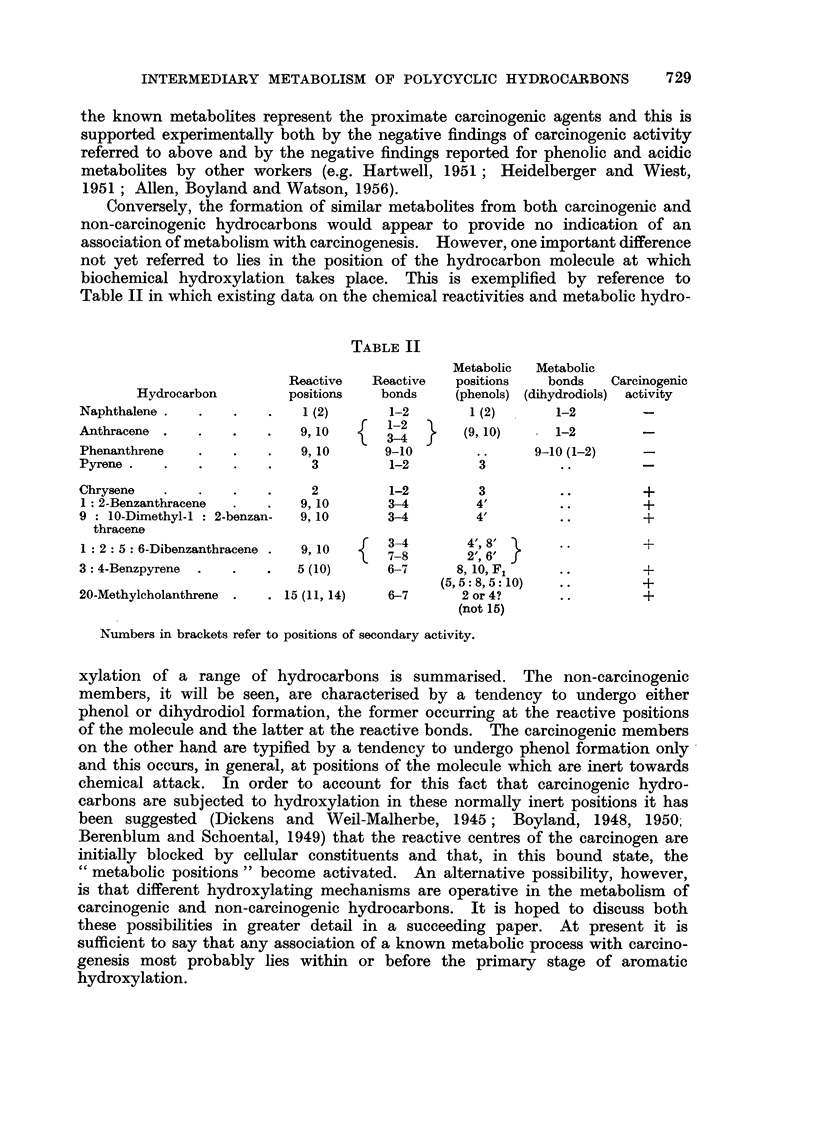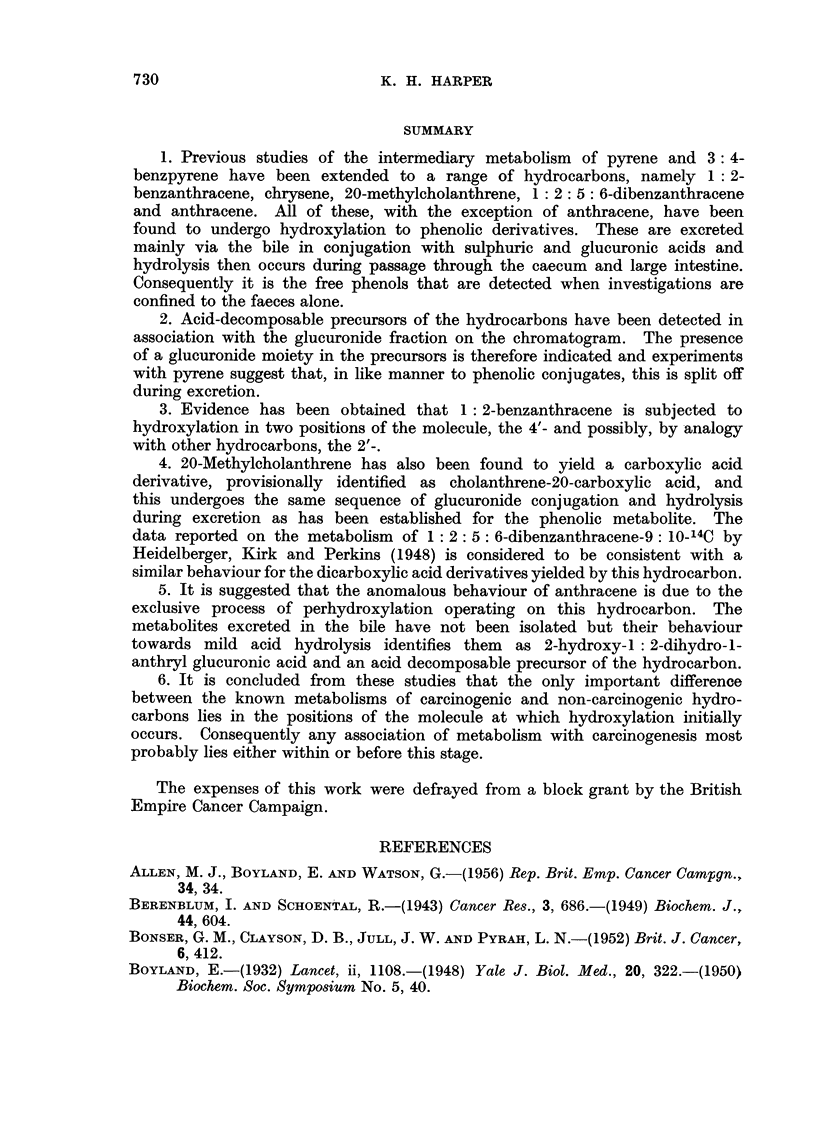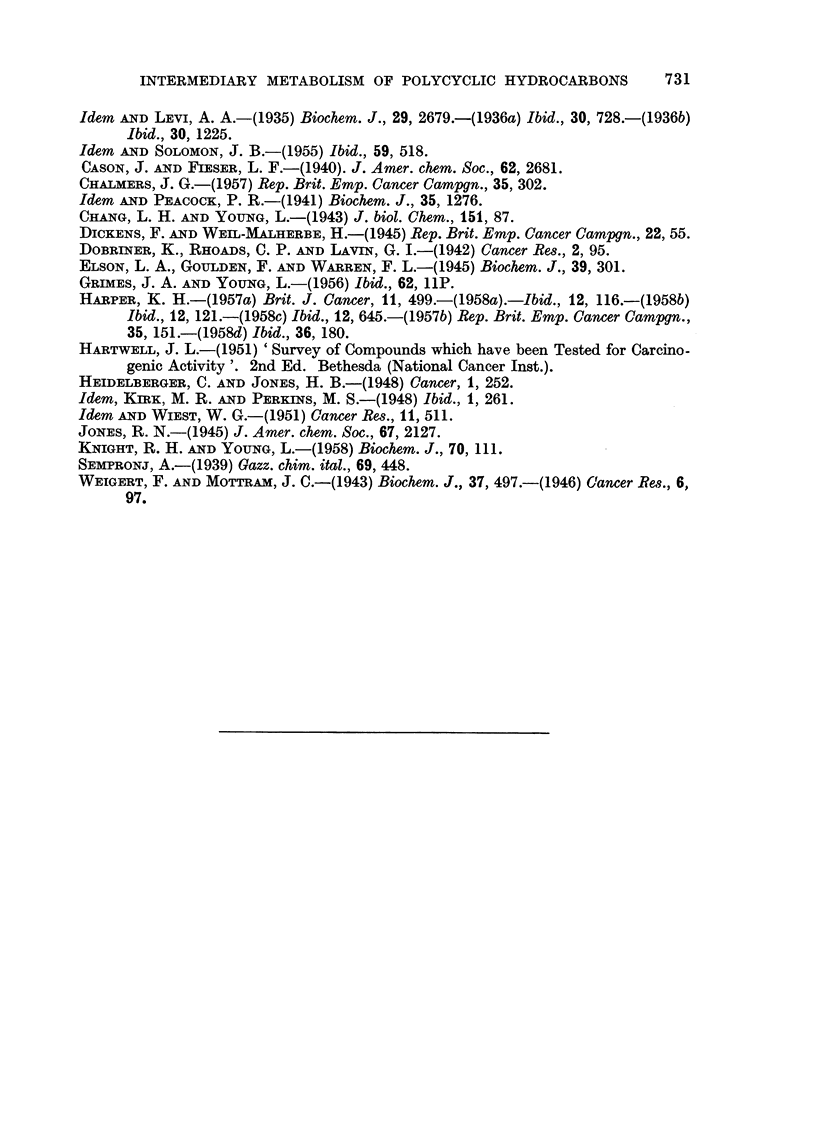# The Intermediary Metabolism of Polycyclic Hydrocarbons

**DOI:** 10.1038/bjc.1959.81

**Published:** 1959-12

**Authors:** K. H. Harper


### THE INTERMEDIARY METABOLISM OF POLYCYCLIC HYDROCARBONS

K. H. HARPER

*From the Department of Cancer Research, Mount Vernon Hospital and the Radium Institute, Northwood, Middlesex*

Received for publication October 31, 1959

PREVIOUS studies of the metabolism of pyrene and 3 : 4-benzpyrene have been reported (Harper, 1957*a*, 1958*a*, 1958*b*, 1958*c*). This work confirmed the earlier finding of Weigert and Mottram (1946) that, following injection of 3 : 4-benzpyrene into mice, two metabolic fractions, designated as $X_1$ and $X_2$, are excreted in the bile and undergo conversion to phenolic derivatives during passage through the intestine. These two fractions were identified respectively as sulphuric acid ($X_1$) and glucuronic acid ($X_2$) esters of fully aromatic benzpyrenols (Harper, 1958*b*, 1958*c*) and a similar sequence of conjugation and hydrolysis was established for the 3-pyrenol metabolite of pyrene (Harper, 1958*a*, 1958*c*). Also associated with the glucuronide fractions yielded by both pyrene and 3 : 4-benzpyrene was an acid-decomposable precursor of the parent hydrocarbon.

This work has now been extended to a range of hydrocarbons, namely 1 : 2-benzanthracene, chrysene, 20-methylcholanthrene, 1 : 2 : 5 : 6-dibenzanthracene and anthracene, utilising the same extraction and analytical procedures. Full details of these are to be found in the above publications.

### MATERIALS AND METHODS

20-Methylcholanthrene (Hoffmann-La-Roche and Co. Ltd.) and anthracene (B.D.H. Ltd.) were used as purchased ; the other hydrocarbons were purified by chromatography on alumina from benzene and cyclohexane followed by fractional crystallisation from ethanol, aq. ethanol or benzene. Colloidal solutions of the hydrocarbons were prepared by the method of Boyland (1932). In the case of 1 : 2 : 5 : 6-dibenzanthracene and chrysene however it was found necessary to heat both acetone solution and water to 50° C. prior to mixing.

Strong A mice, in batches of about twenty, were injected intravenously with 0·5 mg. of colloidal hydrocarbon and the distribution of metabolites within the internal organs of the body was then investigated using the same general methods of extraction and chromatographic separation previously reported for pyrene and 3 : 4 benzpyrene. A modification adopted in the case of faecal metabolites however was as follows.

The freshly voided faeces were extracted into xylene via acetone (Harper, 1957*a*) and the xylene passed through a column of silica gel (100/200 mesh) for the removal of acidic metabolites and much of the colouring matter. The filtrate from the column was evaporated under reduced pressure, the residue extracted repeatedly with boiling cyclohexane and the cyclohexane chromatographed on silica gel. Any phenolic metabolite of the hydrocarbon was then retained as a blue fluorescent zone on the column from which it was readily eluted either with ethanol or with cyclohexane/benzene mixtures. This procedure was preferred to chromatography on alumina as used in previous studies owing to the difficulty experienced in removing small amounts of phenolic derivatives from this adsorbent.

4′-Hydroxy-1 : 2-benzanthracene (4′-benzanthrol) was synthesised by the method of Sempronj (1939). 4′-Methoxy-1 : 2-benzanthracene was prepared from this by methylation using dimethyl sulphate and excess sodium hydroxide, the reaction being continued until the bright golden yellow fluorescence of the solution had disappeared.

1-Anthrol was prepared by reduction of 9 : 10-anthraquinone-1-sulphonic acid followed by alkali fusion.

The reference conjugates, 4′-benzanthryl glucuronide and sulphate and 1-anthryl glucuronide and sulphate, were isolated from the bile and small intestines of mice injected with the parent phenols (cf. Harper, 1958*c*).

### RESULTS

Three hydrocarbons, 1 : 2-benzanthracene, chrysene and 20-methylcholanthrene, yielded well defined blue fluorescent $X_1$ and $X_2$ type metabolites. Excretion was mainly via the bile and hydrolysis to phenolic derivatives and, in the case of 20-methylcholanthrene, also to an acidic derivative, occurred in the caecum and large intestine. Blue fluorescent $X_1$ and $X_2$ type metabolites were also isolated from the bile and small intestine after injection with dibenzanthracene but the yields of these were greatly reduced by comparison with the other hydrocarbons. In marked contrast to this behaviour, however, was the failure of anthracene to yield any material that was recognisable as a metabolite under the same conditions of extraction and analysis.

The $X_1$ type metabolites were readily hydrolysed by cold dilute mineral acid and by takadiastase. $\beta$-Glucuronidase was without effect. The $X_2$ type metabolites on the other hand, were unaffected by treatment with mineral acid in the cold but hydrolysis occurred on prolonged heating under nitrogen with strong acid (6N HCl). Rapid hydrolysis also occurred on incubation with $\beta$-glucuronidase but this effect was nullified in the presence of $10^{-2}$ M boiled saccharate solution.

In view of these properties, and by analogy with the behaviour of the conjugates of 3-pyrenol and benzpyrenols (Harper, 1958*c*), the two metabolic fractions were concluded to be sulphuric and glucuronic acid esters respectively. The essential problem therefore was the identification of the hydrocarbon moieties of the conjugates.

### (*A*) 1 : 2-*Benzanthracene*

The identification of the phenolic derivative of 1 : 2-benzanthracene excreted in the faeces of rats and mice was achieved by methylation and spectroscopic comparison with known methoxy-benzanthracenes (Berenblum and Schoental, 1943). On this basis the 4′-methoxy derivative was selected as being identical with the methylated metabolite although the presence of absorbing impurity limited comparison to the long wave systems (above 300 m$\mu$) of the absorption spectra. A characteristic feature of the spectrum was a band at 391·5 m$\mu$ in hexane but, perhaps surprisingly, the graphical data reported for the methylated metabolite does not include the sharp prominent maximum at 311·5 mμ (Fig. 2).

In the present work attempts have been made to confirm this identification by comparison of the conjugated and free phenolic metabolite with the synthetic analogues derived from 4′-benzanthrol.

(a) *The conjugated metabolites*

The physical and chemical properties of the $X_1$ and $X_2$ type metabolites were respectively identical with those of 4′-benzanthryl sulphate and glucuronide.

FIG. 1.—Absorption spectra in ethanol.

1. $X_1$ type metabolite of benzanthracene (*b* ordinates).
2. 4′-benzanthryl sulphate (*a* ordinates).
3. 4′-benzanthryl glucuronide (*c* ordinates).
4. $X_2$ type metabolite of benzanthracene (*c* ordinates).

The one exception was observed with the $X_2$ type metabolite for, on acid hydrolysis, a small amount of free 1 : 2-benzanthracene was liberated in addition to a phenolic derivative. The presence of an acid-decomposable precursor of the hydrocarbon was therefore indicated.

The absorption spectra of the two sets of conjugates are recorded in Fig. 1. The resemblance between the two types of spectra is at once apparent and provides confirmatory evidence of the conjugated nature of the hydrocarbon metabolites. In Table I however are recorded the detailed positions of the absorption bands and reference to this data reveals that slight differences exist in the location of certain bands. (Failure to record a band at or near 308 m$\mu$ in the spectrum of the $X_2$ type metabolite is possibly due to the presence of absorbing impurity. This was in fact present in one sample of the metabolite isolated from stored bile.)

A possible explanation of these differences is that the hydrocarbon metabolites consist predominantly of the conjugates of 4′-benzanthrol but that smaller amounts of other conjugated derivatives are also present. In the case of the glucuronide fraction this could be the hydrocarbon precursor but, as the sulphate yields only a phenolic fraction on hydrolysis, the presence of an additional benzanthrol is indicated.

(b) *The phenolic metabolite*

The phenolic derivative of 1 : 2-benzanthracene isolated from the faeces could not be obtained free from absorbing impurity and this prevented complete characterisation by absorption spectroscopy. In the long wave region above 320 m$\mu$ however, bands characteristic of 4′-benzanthrol were present and consistent with this behaviour was the bright golden yellow fluorescence exhibited by the metabolite in sodium hydroxide. In view of the possibility of an additional benzanthrol being present the metabolite was subjected to methylation and chromatographic fractionation in the manner described for methoxybenzpyrenes (Harper, 1958*b*). No marked differences were observed in the spectra of the fractions thus obtained and, on pooling and concentrating *in vacuo*, the absorption spectrum shown in Fig. 2 was recorded. Also shown in Fig. 2 is the spectrum of the phenolic derivative liberated from the sulphate conjugate by mild acid hydrolysis and the two are compared respectively with those of 4′methoxy- and 4′-hydroxy-1 : 2-benzanthracene. The detailed positions of the absorption bands are given in Table I.

TABLE I

| Absorption bands in ethanol (m $\mu$) | | | | Absorption bands in hexane (m $\mu$) | | | |
|---|---|---|---|---|---|---|---|
| $BAX_1$ | 4′-BA-S | $BAX_2$ | 4′-BA-G | BA-OH | 4′-OH | BA-OMe | 4′-OMe |
| — | — | — | — | 226 | 230 | 228 | 231 |
| [246–248] | [244–248] | — | 244–246 | — | 245 | 246 | 245–246 |
| 252 | 254 | — | 254 | 253 | 253·5 | 250–254 | 253·5 |
| — | — | — | — | [262] | — | — | — |
| 270 | 272 | — | — | [270–272] | — | — | — |
| 280 | 282 | — | [284–286] | [278–280] | 277 | [280–282] | 277 |
| 290–291 | 292 | 288 | 288 | 286 | 286 | 287–288 | 287·5 |
| — | — | — | — | 297 | 297 | — | 299 |
| — | — | — | 308 | — | 309.5 | — | 311·5 |
| 324 | 326 | — | 328 | [320–324] | 326–327 | [326–328] | 327 |
| — | — | — | — | 337 | — | — | — |
| 342 | 342 | [338–342] | 342 | [340–341] | 342 | 342 | 342·5 |
| — | — | — | 354–356 | 354–355 | 353 | 354 | 352–353 |
| 358 | 359 | [358–362] | 358–360 | — | 359 | [358–360] | 359–360 |
| — | 366–368 | — | 370 | 372 | 371–372 | 370–372 | 371 |
| — | — | — | — | — | 382 | — | 381 |
| 388 | 389 | 392 | 391–392 | 394 | 392 | 392 | 391.5 |

$BAX_1$ and $BAX_2$ = $X_1$ and $X_2$ metabolites of 1 : 2-benzanthracene.
4′-BA-S and 4′-BA-G = sulphuric (S) and glucuronic (G) esters of 4′-benzanthrol.
BA-OH = free phenolic component of the sulphate conjugate ($BAX_1$).
BA-OMe = methylated faecal metabolite.
4′-OH and 4′-OMe = free (– OH) and methylated (–OMe) 4′-benzanthrol.

Reference to this data reveals that, above 320 m$\mu$, the spectra of the methylated metabolite and 4′-methoxy-1 : 2-benzanthracene are very similar and is in agreement with the conclusion of Berenblum and Schoental (1943) that hydroxylation of the benzanthracene nucleus occurs in the 4′-position. It will be recalled that this wave length region only was utilised by these workers for their analysis. In the present work however, the full absorption spectra have been recorded and below 320 m$\mu$ it is at once apparent that marked differences exist. The major band of 4′methoxy-1 : 2-benzanthracene at 287 m$\mu$ is present

FIG. 2.—Absorption spectra in hexane.

1. Phenolic component of the $X_1$ type metabolite of benzanthracene (*b* ordinates).
2. Methylated faecal metabolite (*b* ordinates).
3. 4′-benzanthrol (*a* ordinates).
4. 4′-methoxybenzanthracene (*c* ordinates).

in the " metabolic " spectrum but a conspicuous feature is the absence of any band, other than a slight inflection, at 311·5 m$\mu$. The latter is a prominent feature of the 4′- substituted derivatives and is readily detected in all metabolic studies with these compounds. It is doubtful therefore that failure to record this band is due to the presence of masking impurity of natural origin : A possible explanation is that the presence of an additional related methoxylated benzanthracene interferes with the absorption of the 4′-methoxy derivative in this region although chromatographic fractionation revealed no evidence of this. Support for this proposal however is provided by the spectrum of the free phenol liberated from the sulphate conjugate. This is similar to that of 4′-benzanthrol in its general appearance but differences exist in both short and long wave regions. An unknown phenolic derivative with bands at 226, 286, 297, 320–324,

337, 354–355, 372, and 394 m$\mu$ would indeed appear to represent the major component of the mixture.

In conclusion, therefore, it can be said that the evidence obtained in this work is consistent with the view that a mixture of phenolic derivatives is formed as a result of the biological hydroxylation of 1 : 2-benzanthracene in the mouse. One of them is 4'-benzanthrol, the other is neither 3-benzanthrol (Jones, 1945) nor 9 : 10-dihydroxy-1 : 2 : -benzanthracene (Berenblum and Schoental, 1943) and is as yet unidentified. By analogy with other hydrocarbons however it may logically be expected to be the 2'-hydroxy derivative.

FIG. 3.—Absorption spectra in ethanol.

1. Phenolic metabolite of chrysene (*b* ordinates).
2. $X_1$ type metabolite of chrysene (*a* ordinates).
3. $X_2$ type metabolite of chrysene (*a* ordinates).

### (*B*) *Chrysene*

The phenolic derivative excreted in the faeces after intraperitoneal injection of chrysene in the rat was identified by Berenblum and Schoental (1949) as 3-chrysenol. Attempts to confirm this in the present work with mice have been only partially successful owing to the low yield obtained and difficulty experienced in purification. The experiments have shown however that the two conjugated fractions yield what appears to be the same chrysenol on hydrolysis with takadiastase and $\beta$-glucuronidase respectively and that a small amount of free chrysene is also liberated from the glucuronide fraction on hot acid hydrolysis.

The absorption spectra of the metabolites (Fig. 3), although ill-defined, are consistent with a fully aromatic chrysenoid configuration. The conjugated metabolites are therefore concluded to be (3)-chrysenyl sulphate and glucuronide respectively.

(*C*) 20-*Methylcholanthrene*

This hydrocarbon (I) was of particular interest in its divergence from the fully aromatic configurations so far studied. Although existing evidence on the

I

metabolism of this compound suggested that hydroxylation occurs on the aromatic benzanthracene nucleus (Cason and Fieser, 1940; Dobriner, Rhoads and Lavin, 1942), general metabolic considerations were in favour of both phenol

Fig. 4.—Absorption spectra in ethanol.

1. Carboxylic acid metabolite of 20-methylcholanthrene (*a* ordinates).
2. Phenolic metabolite of 20-methylcholanthrene (*a* ordinates).
3. $X_1$ type metabolite of 20-methylcholanthrene (*b* ordinates).
4. $X_2$ type metabolite of 20-methylcholanthrene (c ordinates).

and carboxylic acid formation. The latter may be expected to arise from the oxidation of the attached methyl group—cf. the metabolism of 2-methylnaphthalene (Grimes and Young, 1956)—or from fission of the 15–16 bond—cf. the metabolism of acenaphthene (Chang and Young, 1943). This theoretical prediction has been borne out experimentally for two methylcholanthrene derivatives, one phenolic and the other acidic, have been isolated in this work. These were absent from the bile and small intestine but were present in the caecum, large intestine and faeces.

The phenolic derivative possessed normal chromatographic behaviour and the typical fluorescence colour change from blue to yellow occurred on the addition of sodium hydroxide to its ethanolic solution. The absorption spectrum shown in Fig. 4 possessed bands at 284, 294, 304–305, 344–346, [352], 363 [378–380] and 398 m$\mu$. It has not been possible to identify it but, by analogy with other hydrocarbons, it may logically be expected to be either the 2- or 4-hydroxy derivative.

The acidic derivative was not adsorbed on silica gel from xylene or benzene but was tightly held as a narrow blue fluorescent zone at the surface of an alumina column. Elution was achieved with ethanol containing 1 per cent hydrochloric acid, ethanol by itself being ineffective. A solution of the metabolite in this mixture possessed a yellow fluorescence which changed to bright blue on making alkaline with sodium hydroxide. Its acidic nature was further emphasised by its solubility in dilute sodium bicarbonate. In view of these properties the metabolite was concluded to be a carboxylic acid derivative. As the absorption spectrum shown in Fig. 4, with bands at 284, 295, 302, 312, 334, 348–350, 364–366, 378–380 and 402 m$\mu$, establishes the integrity of the aromatic benzanthracene nucleus, three possible structures for the metabolite are :

(*a*) Cholanthrene-20-carboxylic acid (II), arising from oxidation of the methyl group.

(*b*) 6-Methyl-1 : 2-benzanthracene-5 : 10-dicarboxylic acid (III), arising from fission at the 15–16 bond.

or

(*c*) 1 : 2-Benzanthracene-5 : 6 : 10-tricarboxylic acid (IV), arising from a combination of these.

II III IV

If the metabolite is either the di-(III) or tri-(IV) carboxylic acid, then anhydride formation is theoretically possible. This was tested by heating the compound at 200° C. for ten minutes. After cooling and dissolving in ethanol no difference in its chromatographic behaviour and absorption spectrum was observed. A tentative conclusion therefore is that the metabolite is cholanthrene-20-carboxylic acid (II).

The absence of the free phenol and carboxylic acid from the gall bladder and small intestine indicated their formation from the conjugated metabolites present in these organs. This was confirmed on enzymatic hydrolysis of the conjugates when the sulphate fraction yielded free phenol alone whilst the glucuronide fraction yielded a mixture of phenol and carboxylic acid. In the latter case advantage was taken of the differential solubilities of the derivatives in sodium bicarbonate to effect a ready separation of the two components from solution in ether. The same behaviour was observed on acid hydrolysis under nitrogen but a further phenomenon recorded in this instance was the liberation of a trace amount of 20-methylcholanthrene from the glucuronide fraction.

The absorption spectra of the conjugated fractions, shown in Fig. 4, establish that the aromatic benzanthracene nucleus is intact. It is concluded from this work therefore that the $X_1$ type metabolite is a sulphuric acid ester of a phenolic derivative of 20-methylcholanthrene whilst the $X_2$ type contains a mixture of glucuronic acid esters of the same phenol and cholanthrene-(20)-carboxylic acid together with an acid-decomposable precursor of the hydrocarbon. Indications of the presence of other water soluble metabolites have also been obtained but these have not been pursued further.

### (*D*) 1 : 2 : 5 : 6-*Dibenzanthracene*

A phenolic derivative, 4′ : 8′-dihydroxy-1 : 2 : 5 : 6-dibenzanthracene, was the first metabolite of 1 : 2 : 5 : 6-dibenzanthracene to be identified (Cason and Fieser, 1940 ; Dobriner, Rhoads and Lavin, 1942). Subsequent investigation by Heidelberger and his collaborators in the United States established that the metabolism of this hydrocarbon is accompanied by extensive degradation of the aromatic nucleus (Heidelberger and Jones, 1948 ; Heidelberger, Kirk and Perkins, 1948) and consistent with this finding is the low yield of fluorescent material which has been isolated in the present work. Owing to the small amounts of metabolites obtained it has not been possible to characterise the compounds individually by absorption spectroscopy although a mixture of the conjugated derivatives extracted from bile possessed absorption indicative of an intact dibenzanthracene nucleus.

Enzymatic hydrolysis of the two conjugated fractions as described resulted in the liberation of a phenolic derivative as adjudged by the fluorescence colour change from blue to yellow occurring on the addition of sodium hydroxide. It was concluded from this behaviour therefore that a phenolic derivative, presumably 4′ : 8′-dihydroxy-dibenzanthracene, is excreted via the bile in conjugation with sulphuric and glucuronic acids. The conjugates could not be detected within the liver or kidney and were absent from the caecum and large intestine. Free phenol was present in the latter organs, however, suggesting that hydrolysis of the conjugates occurs at this site.

These findings are supported by the data reported by Heidelberger, Kirk and Perkins (1948) on the excretion of $^{14}C$-labelled dibenzanthracene following intravenous injection in the mouse. The extraction procedure adopted by these workers enabled the radioactivity of the material under investigation to be split into four fractions which they considered to contain respectively :

(1) Unchanged hydrocarbon.
(2) Unconjugated material.
(3) Extremely water soluble organic substances together with some less soluble material which was conjugated with water solubilising groups.
(4) A solid residue which was partly soluble in water.

Analysis of the data obtained respectively from bile and faeces shows that, during passage through the intestine, there is a transfer of radioactivity from fraction 3, the major component of the biliary activity, to fractions 2 and 4. The increase in fraction 2 is most pronounced, from 0·7 per cent in the bile to 14 per cent in the faeces, suggesting that unconjugated derivatives in the faeces are derived from conjugated compounds excreted in the bile.

It was further shown by these workers that fraction 2 of the faeces contains a mixture of phenolic, acidic and neutral components so that the same sequence of conjugation and hydrolysis presumably occurs with the dicarboxylic acid degradation products as has been established for cholanthrene-(20)-carboxylic acid in the present work.

### (*E*) *Anthracene*

The metabolism of anthracene was studied by Boyland and Levi (1935, 1936*a*, 1936*b*) who reported the excretion in the urine of a perhydroxylated derivative, 1 : 2-dihydroxy-1 : 2-dihydro-anthracene, both free and conjugated with glucuronic acid, an unidentified acid-decomposable precursor of the hydrocarbon and 1-anthrylmercapturic acid. The latter has since been shown by Knight and Young (1958) to arise during extraction from the action of mineral acid upon a precursor designated by the general term " premercapturic acid ".

The excretion of metabolites in the bile has also been reported (Chalmers and Peacock, 1941 ; Chalmers, 1957) but these have not been identified.

The present experiments with anthracene have shown that $X_1$ and $X_2$ type derivatives, analogous to those yielded by the other hydrocarbons studied in this series, are not formed during the metabolism of this compound. The isolation of such compounds, i.e. 1-anthryl sulphuric and glucuronic acids, from the bile following injection of 1-anthrol, under the same conditions of extraction, established that failure to detect these derivatives during metabolism of the parent hydrocarbon was not due to defects of the extraction process.

The situation was found to be quite different however when the bile and aqueous extracts of the duodenum and small intestine were first subjected to mild acid hydrolysis. Under these conditions an $X_2$ type derivative, identical with 1-anthrylglucuronic acid, and free anthracene were readily extractable from the acid solutions. A blue fluorescent chloroform soluble fraction was also present but this was not investigated further.

As the 2-hydroxy-1 : 2-dihydro- 1 -anthrylglucuronic acid excreted in the urine was found by Boyland and Levi (1936*a*) to break down readily under the influence of acid to yield 1-anthrylglucuronic acid, it was concluded from the above behaviour that this perhydroxylated conjugate is also excreted in the bile together with an acid-decomposable precursor of anthracene and possibly a " premercapturic acid ".

### DISCUSSION

The investigations reported in this and previous publications were part of a programme designed to determine the role, if any, played by the metabolism of polycyclic hydrocarbons in induced carcinogenesis. Should the formation of the metabolites be implicated in the carcinogenic mechanism or themselves be the initiators of it—cf. the mode of action of 2-naphthylamine (Bonser, Clayson,

Jull and Pyrah, 1952)—it was considered necessary that the following requirements be fulfilled :

(*a*) They should be present within the tissues of the body where the carcinogenic effect is applied.

(*b*) The same type of metabolites should be formed from all carcinogenic but not non-carcinogenic hydrocarbons (quantitative factors are ruled out by the fact that the carcinogenic members exert their effect when present in only trace amounts).

(*c*) If themselves the proximate carcinogenic agents then this activity should be detectable by normal laboratory methods of testing.

Requirement (*a*) was met by the intermediate $X_1$ and $X_2$ metabolites of 3 : 4-benzpyrene previously isolated by Weigert and Mottram (1943). Extension of this work to a range of hydrocarbons has now shown that similar intermediates are formed from the non-carcinogenic pyrene, the weakly carcinogenic chrysene and 1 : 2-benzanthracene and the strongly carcinogenic 1 : 2 : 5 : 6-dibenzanthracene and 20-methylcholanthrene. These intermediates have been found to possess a common structure, i.e. phenolic derivatives conjugated with sulphuric acid ($X_1$) and glucuronic acid ($X_2$), and the association of acid-decomposable precursors of the hydrocarbons with the $X_2$ fractions suggests that these too may contain a glucuronide conjugated hydroxyl group. Indeed, the experiments with pyrene (Harper, 1957*a*, 1958*a*) suggest that, in like manner to the phenolic conjugates, the glucuronide moiety of the precursors is split off during excretion for the pyrene precursor in the faeces was then found in association with the neutral quinone fraction on the chromatogram. Consistent with this behaviour would be the presence of an $\alpha : \beta$-dihydro-$\alpha$-hydroxy configuration similar to that established for the precursor of naphthalene (Boyland and Solomon, 1955).

The metabolisms of 1 : 2 : 5 : 6-dibenzanthracene and 20-methylcholanthrene are further complicated by the formation of carboxylic acid derivatives but this does not appear to be a general feature of the carcinogenic series.

The anomalous behaviour of anthracene must be attributed to the exclusive process of perhydroxylation, as opposed to that of hydroxylation, operating on this hydrocarbon. As dihydrodiols appear to undergo conjugation exclusively with glucuronic acid the failure of anthracene to bring about any significant increase in the level of ethereal sulphate excretion in the urine (Elson, Goulden and Warren, 1945) is consistent with the finding that phenol formation does not occur.

Only in the case of 3 : 4-benzpyrene has it been possible to isolate sufficient of the intermediate metabolites for carcinogenicity testing. These have been tested singly, in combination with each other and in combination with a co-carcinogen, croton oil, but in no case has malignant tumour formation been observed (Harper, 1957*b*, 1958*d*). Other hydrocarbon derivatives tested for activity include the hypothetical metabolites of anthracene, 1- and 2-anthrol. These were found to be inactive suggesting that the non-carcinogenic nature of this hydrocarbon is not due to the protective influence of dihydrodiol formation (unpublished data).

The overall conclusion from this work therefore is that there appears to be no specific difference in the chemical nature of the metabolites derived respectively from carcinogenic and non-carcinogenic hydrocarbons. It is unlikely then that the known metabolites represent the proximate carcinogenic agents and this is supported experimentally both by the negative findings of carcinogenic activity referred to above and by the negative findings reported for phenolic and acidic metabolites by other workers (e.g. Hartwell, 1951 ; Heidelberger and Wiest, 1951 ; Allen, Boyland and Watson, 1956).

Conversely, the formation of similar metabolites from both carcinogenic and non-carcinogenic hydrocarbons would appear to provide no indication of an association of metabolism with carcinogenesis. However, one important difference not yet referred to lies in the position of the hydrocarbon molecule at which biochemical hydroxylation takes place. This is exemplified by reference to Table II in which existing data on the chemical reactivities and metabolic hydro-

TABLE II

| Hydrocarbon | Reactive positions | Reactive bonds | Metabolic positions (phenols) | Metabolic bonds (dihydrodiols) | Carcinogenic activity |
|---|---|---|---|---|---|
| Naphthalene | 1 (2) | 1–2 | 1 (2) | 1–2 | — |
| Anthracene | 9, 10 | 1–2<br>3–4 | (9, 10) | 1–2 | — |
| Phenanthrene | 9, 10 | 9–10 | .. | 9–10 (1–2) | — |
| Pyrene | 3 | 1–2 | 3 | .. | — |
| Chrysene | 2 | 1–2 | 3 | .. | + |
| 1 : 2-Benzanthracene | 9, 10 | 3–4 | 4′ | .. | + |
| 9 : 10-Dimethyl-1 : 2-benzanthracene | 9, 10 | 3–4 | 4′ | .. | + |
| 1 : 2 : 5 : 6-Dibenzanthracene | 9, 10 | 3–4<br>7–8 | 4′, 8′<br>2′, 6′ | .. | + |
| 3 : 4-Benzpyrene | 5 (10) | 6–7 | 8, 10, $F_1$ | .. | + |
| | | | (5, 5 : 8, 5 : 10) | .. | + |
| 20-Methylcholanthrene | 15 (11, 14) | 6–7 | 2 or 4?<br>(not 15) | .. | + |

Numbers in brackets refer to positions of secondary activity.

xylation of a range of hydrocarbons is summarised. The non-carcinogenic members, it will be seen, are characterised by a tendency to undergo either phenol or dihydrodiol formation, the former occurring at the reactive positions of the molecule and the latter at the reactive bonds. The carcinogenic members on the other hand are typified by a tendency to undergo phenol formation only and this occurs, in general, at positions of the molecule which are inert towards chemical attack. In order to account for this fact that carcinogenic hydrocarbons are subjected to hydroxylation in these normally inert positions it has been suggested (Dickens and Weil-Malherbe, 1945 ; Boyland, 1948, 1950; Berenblum and Schoental, 1949) that the reactive centres of the carcinogen are initially blocked by cellular constituents and that, in this bound state, the " metabolic positions " become activated. An alternative possibility, however, is that different hydroxylating mechanisms are operative in the metabolism of carcinogenic and non-carcinogenic hydrocarbons. It is hoped to discuss both these possibilities in greater detail in a succeeding paper. At present it is sufficient to say that any association of a known metabolic process with carcinogenesis most probably lies within or before the primary stage of aromatic hydroxylation.

SUMMARY

1. Previous studies of the intermediary metabolism of pyrene and 3 : 4-benzpyrene have been extended to a range of hydrocarbons, namely 1 : 2-benzanthracene, chrysene, 20-methylcholanthrene, 1 : 2 : 5 : 6-dibenzanthracene and anthracene. All of these, with the exception of anthracene, have been found to undergo hydroxylation to phenolic derivatives. These are excreted mainly via the bile in conjugation with sulphuric and glucuronic acids and hydrolysis then occurs during passage through the caecum and large intestine. Consequently it is the free phenols that are detected when investigations are confined to the faeces alone.

2. Acid-decomposable precursors of the hydrocarbons have been detected in association with the glucuronide fraction on the chromatogram. The presence of a glucuronide moiety in the precursors is therefore indicated and experiments with pyrene suggest that, in like manner to phenolic conjugates, this is split off during excretion.

3. Evidence has been obtained that 1 : 2-benzanthracene is subjected to hydroxylation in two positions of the molecule, the 4′- and possibly, by analogy with other hydrocarbons, the 2′-.

4. 20-Methylcholanthrene has also been found to yield a carboxylic acid derivative, provisionally identified as cholanthrene-20-carboxylic acid, and this undergoes the same sequence of glucuronide conjugation and hydrolysis during excretion as has been established for the phenolic metabolite. The data reported on the metabolism of 1 : 2 : 5 : 6-dibenzanthracene-9 : 10-$^{14}$C by Heidelberger, Kirk and Perkins (1948) is considered to be consistent with a similar behaviour for the dicarboxylic acid derivatives yielded by this hydrocarbon.

5. It is suggested that the anomalous behaviour of anthracene is due to the exclusive process of perhydroxylation operating on this hydrocarbon. The metabolites excreted in the bile have not been isolated but their behaviour towards mild acid hydrolysis identifies them as 2-hydroxy-1 : 2-dihydro-1-anthryl glucuronic acid and an acid decomposable precursor of the hydrocarbon.

6. It is concluded from these studies that the only important difference between the known metabolisms of carcinogenic and non-carcinogenic hydrocarbons lies in the positions of the molecule at which hydroxylation initially occurs. Consequently any association of metabolism with carcinogenesis most probably lies either within or before this stage.

The expenses of this work were defrayed from a block grant by the British Empire Cancer Campaign.

### REFERENCES

Allen, M. J., Boyland, E. and Watson, G.—(1956) *Rep. Brit. Emp. Cancer Campgn.*, **34**, 34.

Berenblum, I. and Schoental, R.—(1943) *Cancer Res.*, **3**, 686.—(1949) *Biochem. J.*, **44**, 604.

Bonser, G. M., Clayson, D. B., Jull, J. W. and Pyrah, L. N.—(1952) *Brit. J. Cancer*, **6**, 412.

Boyland, E.—(1932) *Lancet*, ii, 1108.—(1948) *Yale J. Biol. Med.*, **20**, 322.—(1950) *Biochem. Soc. Symposium* No. 5, 40.

*Idem* AND LEVI, A. A.—(1935) *Biochem. J.*, **29**, 2679.—(1936*a*) *Ibid.*, **30**, 728.—(1936*b*) *Ibid.*, **30**, 1225.
*Idem* AND SOLOMON, J. B.—(1955) *Ibid.*, **59**, 518.
CASON, J. AND FIESER, L. F.—(1940). *J. Amer. chem. Soc.*, **62**, 2681.
CHALMERS, J. G.—(1957) *Rep. Brit. Emp. Cancer Campgn.*, **35**, 302.
*Idem* AND PEACOCK, P. R.—(1941) *Biochem. J.*, **35**, 1276.
CHANG, L. H. AND YOUNG, L.—(1943) *J. biol. Chem.*, **151**, 87.
DICKENS, F. AND WEIL-MALHERBE, H.—(1945) *Rep. Brit. Emp. Cancer Campgn.*, **22**, 55.
DOBRINER, K., RHOADS, C. P. AND LAVIN, G. I.—(1942) *Cancer Res.*, **2**, 95.
ELSON, L. A., GOULDEN, F. AND WARREN, F. L.—(1945) *Biochem. J.*, **39**, 301.
GRIMES, J. A. AND YOUNG, L.—(1956) *Ibid.*, **62**, 11P.
HARPER, K. H.—(1957*a*) *Brit. J. Cancer*, **11**, 499.—(1958*a*).—*Ibid.*, **12**, 116.—(1958*b*) *Ibid.*, **12**, 121.—(1958*c*) *Ibid.*, **12**, 645.—(1957*b*) *Rep. Brit. Emp. Cancer Campgn.*, **35**, 151.—(1958*d*) *Ibid.*, **36**, 180.
HARTWELL, J. L.—(1951) ' Survey of Compounds which have been Tested for Carcinogenic Activity '. 2nd Ed. Bethesda (National Cancer Inst.).
HEIDELBERGER, C. AND JONES, H. B.—(1948) *Cancer*, **1**, 252.
*Idem*, KIRK, M. R. AND PERKINS, M. S.—(1948) *Ibid.*, **1**, 261.
*Idem* AND WIEST, W. G.—(1951) *Cancer Res.*, **11**, 511.
JONES, R. N.—(1945) *J. Amer. chem. Soc.*, **67**, 2127.
KNIGHT, R. H. AND YOUNG, L.—(1958) *Biochem. J.*, **70**, 111.
SEMPRONJ, A.—(1939) *Gazz. chim. ital.*, **69**, 448.
WEIGERT, F. AND MOTTRAM, J. C.—(1943) *Biochem. J.*, **37**, 497.—(1946) *Cancer Res.*, **6**, **97**.